# Late myocardial sequelae of electrical injury

**DOI:** 10.1002/ccr3.3413

**Published:** 2020-10-27

**Authors:** Ofir Koren, Ehud Paz, Ehud Rozner, Muhamad Mahamid, Yoav Turgeman

**Affiliations:** ^1^ Heart Institute Emek Medical Center Afula Israel; ^2^ Bruce Rappaport Faculty of Medicine Technion‐Israel Institute of Technology Haifa Israel; ^3^ Intensive Care Unit Emek Medical Center Afula Israel

**Keywords:** electrical injury, electrocution, out of hospital cardiac arrest, ventricular fibrillation

## Abstract

Electrocution poses serious complications seen mostly at the time of the event. Physicians and patients are usually not aware of the progressive nature and its potentially delayed effect as demonstrated in our case. We believe that a risk stratification model should be designed to guide physicians for proper management.

## Graphical abstract

Electrocution poses serious complications seen mostly at the time of the event. Physicians and patients usually do not aware of the progressive nature and its potentially delayed effect as demonstrated in our case. We believe that a risk stratification model should be designed to guide physicians for proper management.

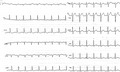

## 1 BACKGROUND

Cardiac involvement in electrical injury is rare yet poses serious manifestations with a high mortality rate. In most cases, symptoms occur immediately after the incident yet, delayed cardiac arrhythmias may occure later and require close monitoring . We present a case of cardiac arrest six hours following an uneventful electrocution. The case emphasizes the potential late sequel to cardiac injury.

Electrical injuries are relatively common and occur mostly at home or as a result of work accidents. Approximately 1000 deaths per year are due to electrical injuries in the United States, with a mortality rate of 3%‐5%.^1^ Cardiac involvement is rare, yet poses the most serious manifestations with high mortality rate.^2^, ^3^, ^4^ In the vast majority of cases, the symptoms occur immediately after the incident, and only in rare cases delayed manifestations are observed.^5^, ^6^ The pathogenesis is not fully understood but in vitro studies and postmortem, autopsies revealed that, in selective patients, electrocution injury progress into permanent scar with late cardiac manifestation as arrhythmia.^7^, ^8^, ^9^, ^10^, ^11^, ^12^, ^13^

## 2 CASE PRESENTATION

We present a 28‐year‐old male heavy smoker, without any known chronic illnesses or family history of cardiac disease or sudden cardiac death. He worked as an exterminator using pesticides in a palm plantation. He was electrocuted by touching 3000 volts of exposed electric wire. He reported pain and new pigmentation in his right hand and left foot. However, he denied losing consciousness, incontinence, chest pain, or palpitation. He did not seek medical care. Six hours later he returned to work and suddenly collapsed with cardiac arrest. His coworkers immediately initiated resuscitation. A few minutes later, a local nurse from a nearby town placed an automated external defibrillator (AED). The AED indicated ventricular fibrillation and 3 DC shocks (200J) were delivered successfully, with the return of spontaneous circulation immediately after. During evacuation, the patient showed signs of respiratory failure; breathed heavily with six breaths per minute and had low oxygen saturation. Several attempts to perform mechanical intubation using 300 mg Ketamine and 20 mg Etomidate given intravenously, failed.

**Figure 1:**
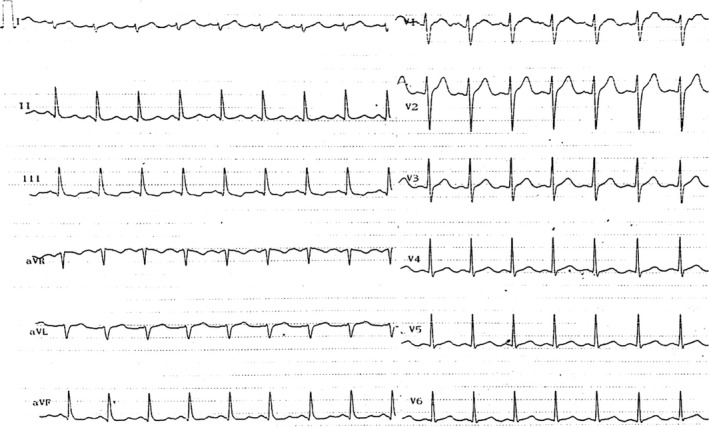
ECG on admission

Upon arrival at the local hospital, he was somnolent with an estimated Glasgow coma scale of 8 and pinpoint pupils. Mechanical ventilation was finally achieved after sedation using intravenous propofol and fentanyl. Arterial blood pressure was 113/60 mm Hg O2 saturation?, and pulse was rhythmic and rapid. Burns were noticed on his right palm and left foot. ECG on admission showed sinus tachycardia, (110‐120 beat/min), right axis deviation, prolonged QT interval (QTc = 520 msec), and inverted T wave in leads III, aVF without ST‐segment deviation (Figure 1). Blood test revealed elevated serum creatinine phosphokinase (840 mg/dL. Normal: <200 mg/dL) with normal serum sodium and potassium. Serum troponin was not measured.

A point‐of‐care echocardiogram, performed by a senior cardiologist, indicated good global systolic function, with an estimated 55% left ventricular ejection fraction. No significant valvular disturbance, nor pericardial effusion or regional wall motion abnormality were noticed. A full‐body CT scan was performed without any significant pathological findings.

Therapeutic hypothermia was initiated for 24 hours, with a target temperature of 34 °C. Two days later, he was able to breathe without oxygen support and could recall the initial events. No serious neurological deficits were noticed. QTc interval return to a normal value of 430 msec and the main axis appeared normal. He was treated mainly with respiratory physiotherapy and was discharged a few days later, fully functioning.

In the following months, the patient complained of anxiety, insomnia, urine incontinence, and palpitations. He denied syncope or near syncope. He was examined by a neurologist and performed an electroencephalogram (EEG) with no pathological finding. The patient was followed up by a cardiologist in the ambulatory clinic. Electrocardiogram and echocardiogram performed two months later revealed no difference compared to previous findings. 24‐hour ECG monitoring (Holter) indicated few isolated ventricular premature beats. Cardiac MRI was not performed.

## 3 DISCUSSION

The most common manifestation of cardiac involvement in electrocution is arrhythmia. The extent of the damage varies from harmless transient sinus tachycardia to fatal ventricular arrhythmia and severe conduction abnormalities which in some cases required permanent pacemaker.^14^

In the vast majority of cases, the onset of injury is immediately after the incident. In rare, cases, as in ours, the onset of the injury may be after several hours, and its effect may last longer—from minutes to several weeks.^15^

Various factors seem to influence the extent of the myocardial involvement such as pre‐existing myocardial injury, the magnitude of energy delivered, the type of current (direct vs alternating), duration of electrocution, and current pathway.^16^

Cardiovascular involvement was found to correlate with the current pathway. The presence of the heart along the current path, as horizontality (hand to hand) or vertically (hand to contralateral foot), increases the likelihood of cardiovascular effects. This can be estimated based on the entrance and exit points.^17^

The mechanism of cardiac arrhythmias triggered by electrocution is not fully understood and mainly based on myocardial biopsy and MRI. It is assumed that delayed arrhythmias are the consequence of changes in the membrane potential of the fibrotic tissue. The injured tissue leads to transient and localized changes in sodium and potassium transport, potassium concentration, and membrane potential across the Na‐K pump.^15^ These changes, as observed by Opie, leads to arrhythmogenic foci with abnormally enhanced automaticity and triggered activity.^18^, ^19^

In our case, we believe that the trigger for the catastrophic arrhythmia was ongoing progressive myocardial injury as a result of electrical and thermal damage alongside the electrocution pathway. No other factors were contributing to arrhythmia other than the electrocution itself.

Appropriate recommendations regarding predisposing risk factors, management, and monitoring of patients who sustained electrical injury have not been well defined, especially due to the rarity of the event and the lack of long‐term follow‐up.^20^, ^21^, ^22^, ^23^

We believe that a risk stratification model should be designed to guide physicians for proper management and close ICU monitoring; taking into consideration the patient clinical characteristics and the likelihood of late cardiac involvement based on objective characteristics of the electrocution event. The role of cardiac MRI should be evaluated for risk stratification both in the acute and the chronic phases and for the prediction of a long‐term adverse cardiovascular event.

## 4 CONCLUSION

Electrical injury causes various arrhythmias, mostly at the time of the incident. Physicians and patients should be aware of the delayed effect of severe arrhythmia following electrocution. Patients should be followed up and monitored for 24 hours when the suspicious of myocardial involvment is high as in the cases of unwitnessed event, exposure to high voltage, current pathway involving central chest, or the present of ECG abnormalities. Physicians should pay attention to the current pathway, assume by noticeable burns. Patients should be followed up for several months by multidiscipline physicians, among them, a cardiologist, neurologist, psychiatrist, and a physiotherapist. Cardiac MRI should be used in acute settings to assess the extent of myocardial injury and during a follow‐up period to predict the risk of a repeated arrhythmic event.

## 5 DATA AVAILABILITY STATEMENT

The datasets used and/or analyzed during the current study are available from the corresponding author on reasonable request.

## CONFLICT OF INTEREST

None declared.

## AUTHOR CONTRIBUTIONS

OK and ER: contributed to the writing, editing, formatting of the main manuscript, and production of the figures. EP and YT: provided care to the patient and revised the manuscript. All authors have contributed and met the criteria for authorship.

## CONSENT FOR PUBLICATION

Consent form was signed by the patient. Any patient‐related data described in the case will be confidential.
